# Xianling Gubao Capsule Prevents Cadmium-Induced Kidney Injury

**DOI:** 10.1155/2021/3931750

**Published:** 2021-09-28

**Authors:** Jian Huang, Xiao-tong Ma, Duo-duo Xu, Bao-jin Yao, Da-qing Zhao, Xiang-yang Leng, Jia Liu

**Affiliations:** ^1^Department of Pharmaceutical Science, Changchun University of Chinese Medicine, Changchun, China; ^2^Jilin Ginseng Academy, Changchun University of Chinese Medicine, Changchun, Jilin 130117, China; ^3^The Affiliated Hospital of Changchun University of Chinese Medicine, Changchun, Jilin 130117, China

## Abstract

Xianling Gubao Capsule (XGC), a kind of capsule preparation of Chinese herbal officially approved for sale by the National Medical Products Administration (NMPA), has the effect of tonifying kidney and strengthening bones. Although the impact of XGC in treating bone diseases has been widely studied, the effect of XGC in kidney injury is unknown yet. The kidney injury model is established by intraperitoneal injection with cadmium chloride (CdCl_2_). Before model establishment, each XGC group was pregavaged with XGC for 10 d. After 10 d, CdCl2 was injected intraperitoneally into the model group and each XGC group, each XGC group continued to be gavaged with XGC for 4 weeks, and the control group was gavaged with equal doses of distilled water once daily. The level of serum urea nitrogen (BUN) and serum creatinine (Cr) is evaluated by kit. The effect of XGC on protecting kidney injury in mice with kidney injury is analyzed by histopathology (HE stain), immunohistochemistry (IHC), and real-time fluorescence quantitative PCR (RT-qPCR). The results show that CdCl_2_ significantly increases the level BUN and Cr in serum and results in remarkable pathological changes in the nephron, including tubule edema, congestion, and necrosis. While oral administration of XGC can significantly decrease BUN and Cr in serum and prevent and protect the kidney from the above injuries. In addition, the protein expression of p-mTOR was remarkably reduced, and the ratio of LC3II/LC3I protein and mRNA was significantly increased in mice with oral administration of XGC. Our findings suggest that XGC can prevent and protect kidney injury by improving the state of renal tubular hyperemia and necrosis and reduce the level of BUN and Cr in cadmium poisoning mice.

## 1. Introduction

Kidney injury is a global public health problem, causing 1.7 million deaths each year. Many reasons can cause kidney injury, such as sepsis, drugs, and toxins. When kidney injury occurs, there are usually tubulointerstitial and glomerular lesions in the tissue [1]. If the patient cannot recover from the kidney injury, it will develop into chronic kidney disease (CKD) and eventually develop into renal failure [2]. The final death caused by chronic renal injury has caused a severe social crisis, so people have carried out a lot of research in this field. There is evidence that autophagy has a protective effect on renal injury [3, 4]. When acute kidney injury (AKI) occurs, autophagy is activated in renal tubular epithelial cells, and blocking autophagy will lead to the deterioration of the disease, while inducing autophagy can reduce the injury [5, 6]. At present, symptomatic treatment and alternative therapy are still the main strategies. Therefore, it is essential to prevent the treatment of diseases other than kidney injury.

As a part of conservative treatment of kidney injury, traditional Chinese medicine and natural product have irreplaceable advantages. XGC is a Chinese herbal capsule preparation officially approved for sale by NMPA in 2002. It is composed of six traditional Chinese herbs—Epimedii Folium (*Epimedium brevicomu* Maxim), Salvia miltiorrhiza Radix Et Rhizoma (*Salvia miltiorrhiza* Bunge), Anemarrhenae Rhizoma (*Anemarrhena asphodeloides* Bunge), Psoraleae Fructus (*Cullen corylifolium* (Linnaeus) Medikus), Dipsaci Radix (*Dipsacus asper* Wallich ex Candolle), and Rehmanniae Radix (*Rehmannia glutinosa* (Gaert.) Libosch. ex Fisch. et Mey). XGC can prevent and cure complications, delay the progressive injury of renal function, protect the residual nephron, and cooperate with other treatment measures to improve patients' symptoms, the quality of life, and prolong life. It affects tonifying the kidney and strengthening bone and can be used to prevent and treat osteopathy, osteoarthritis, and climacteric syndrome caused by renal dysfunction. It was included in China's national catalog of essential medicines in 2009 [7]. Studies have shown that Epimedii Folium, Salvia miltiorrhiza Radix Et Rhizoma, and Rehmanniae Radix in XGC have protective effects on various types of renal function injury, regulate systemic metabolism, improve microinflammatory state, improve renal tubular function, reduce membrane permeability, protect residual renal function, and promote the recovery of renal tissue through multipathway and the multitarget way [8–10]. Natural products have great potential for development, and many studies have been done, especially on kidney injury. Almeer et al. [11] conducted relevant studies on royal jelly. They showed that royal jelly pretreatment exerted a protective effect against Cd-induced nephrotoxicity in mice by facilitating Cd execretion, restoring the oxidant/antioxidant balance, and preventing inflammation and apoptosis.

Cadmium is a heavy metal pollutant, which mainly exists in smelting, electroplating, phosphate fertilizer, and smoke [12, 13]. As the industry develops, heavy metal is a primary pollutant, and cadmium plays a central role in severe kidney injury. Cadmium enters the body in various ways, including water through the digestive tract, food, and air through the respiratory tract. Many studies have used ClCd_2_ intraperitoneal injection to establish the model of kidney injury [1, 14]. Studies have shown that a long-term low concentration of cadmium intake can cause pathological injuries such as edema and hemorrhagic necrosis of the renal tubule [14]. Thus, in this research, we used intraperitoneal injection with CdCl_2_ to establish the kidney injury model and investigated the effect of XGC for preventing mice from cadmium-induced kidney injury.

## 2. Materials and Methods

### 2.1. Animals and Drug

Female C57BL/6 mice weighing 18 ± 22 g were purchased from the Liaoning Chang-sheng Biotechnology Co., Ltd. (Shenyang, China). XGC was purchased from Tongji Tang (Guizhou) Pharmaceutical Co., LTD. The animal experiments were approved by the Ethics Committee of Changchun University of Chinese Medicine (Changchun, China). All animal procedures followed the National Institutes of Health Guide for the Care and Use of Laboratory Animals. All mice were housed at a standard room temperature of 23 ± 2°C and humidity of 55–70% under a 12 h light/dark cycle with access to food and water *ad libitum*.

### 2.2. Experimental Design and Drug Administration

After a week of adaptive feeding, C57BL/6 mice were randomly divided into 5 groups (*n* = 5): control group, model group, XGC low-dose group, XGC medium-dose group, and XGC high-dose group. Before model establishment, XGC groups were intragastric with a corresponding dose of XGC for 10 d, once a day. From day 11, CdCl_2_ (2 mg/kg/d) was injected intraperitoneally into the model group and each XGC group, each XGC group simultaneously continued to gavage XGC once a day for 4 weeks, and the control group was gavaged with equal doses of distilled water once daily. The kidney injury mice model was confirmed with the characteristics of kidney pathology. Model group mice were treated with distilled water; XGC low-dose group mice were treated with XGC at a dosage of 0.182 g/kg/d; XGC middle-dose group mice were treated with XGC at a dosage of 0.364 g/kg/d; XGC high-dose group mice were treated with XGC at a dosage of 0.728 g/kg/d. Besides, the control group mice were treated with distilled water and served as normal control. After 4 weeks of treatment, blood samples were collected from the eye vein by rapidly removing the eyeball. The kidney was also removed, then the left kidney was fixed in 10% neutral formalin for histological analysis, and the right kidney was stored at -80°C for RNA extraction.

### 2.3. Serum Analysis

Concentrations of serum creatinine (Cr) and urea nitrogen (BUN) levels were detected using the corresponding assay kit provided by Nanjing Jiancheng Bioengineering Institute (Nanjing, China). The principle behind the assay kit used for the analysis of BUN was based on the substance produced by the hydrolysis of urea under the action of urease forms a blue substance under the action of phenol chromogenic agent and is determined at 640 nm wavelength. For Cr assay, a reaction occurs between creatinine and peroxidase to produce a purplish red-colored complex, which can be measured using a spectrophotometric plate reader at an optical density of 546 nm.

### 2.4. Pathological and Immunohistochemical Staining Assay

Mice kidneys were fixed in 10% neutral formalin for 48 h and embedded in paraffin. Then, the kidney tissues were cut into 5 *μ*m thick sections. The sections were stained with hematoxylin and eosin and observed under a light microscope (Eclipse Ci-L, Nikon) at 200 and 400 magnification. For the detection of autophagy-related proteins, the prepared kidney sections were blocked with 3% hydrogen peroxide for 15 min to terminate endogenous peroxidase activity, and 10% normal goat serum was added to evenly cover tissue to seal 30 min at room 23-26°C. Afterward, the tissue slices were incubated with primary antibody (Wanleibio, China) mTOR (WL02477, 1 : 200), p-mTOR (WLH3897, 1 : 200), beclin1 (WL02508, 1 : 200), and LC3 (WL01506, 1 : 300) antibody at 4°C for 24 h. The tissues were washed with phosphate-buffered saline and incubated with biotinylated secondary antibody for 50 min. Added diaminobenzidine (DAB) chromogenic solution to cover the tissue evenly, controlled the color development under the microscope, and rinsed with pure water to stop the color development. Nikon DS-F12 (Tokyo, Japan) was used to take microscopy images of all the specimens at 400× magnification.

### 2.5. Real-Time Quantitative Polymerase Chain Reaction (RT-qPCR)

Total RNA of the kidney was extracted using Trizol reagent (Tiangen, China) according to the manufacturer's protocol. The concentration and purity of isolated RNA were determined by BioSpec-nan (Shimadzu, Japan). Total RNA (1 *μ*g) was reversely transcribed into cDNA using FastKing Reverse Transcriptase Kit (Tiangen, China). cDNA samples were used to amplify the target genes using SuperReal PreMix Plus (SYBR Green) kits (Tiangen, China) on an Eppendorf AG 22331 Real-Time PCR system (Eppendorf, Germany). Specific primers (Table 1) were synthesized by Comate, China. GAPDH expression in each sample was included as the internal control. Gene expression was quantitated using the 2^−∆∆Ct^ method.

### 2.6. Western Blot Analysis

Kidney tissue were centrifuged at 12,000 g for 10 min at 4 °C, and the supernatant was used to determine total protein concentration using a BCA protein assay kit. Proteins were separated using polyacrylamide gel electrophoresis and transferred onto polyvinylidene fluoride (PVDF) membranes (Millipore, USA). Equivalent amounts of protein were separated on 10% sodium dodecyl sulfate–polyacrylamide gel electrophoresis and transferred onto polyvinylidene fluoride membranes (Millipore, Danvers, MA, USA). Each membrane was blocked for 1 h using 5% nonfat dry milk, incubated overnight with primary antibodies (dilution 1 : 1000) at 4°C, and then with secondary antibodies (dilution 1 : 10000) for 1 h at room 23-26°C. The primary antibodies involved were against mTOR, p-mTOR (Ser2448), Beclin1, LC3, and *β*-actin (1 : 1000, CST, Boston, MA, USA). The secondary antibodies were anti-rabbit immunoglobulin G (1 : 10000, Proteintech). The membranes were detected by enhanced chemiluminescence (ECL). Data analysis was performed using Image J (NIH Image, Stuttgart, Germany).

### 2.7. Statistical Analysis

The results are expressed as the mean value and standard deviation. The significance of differences was analyzed by analysis of variance. The analysis was performed using one-way analysis of variance (ANOVA) while the Turkey test was applied to determine whether the differences between the groups were significant (Graph Pad Prism v8.2.1). A value of ^∗^*p* < 0.05 is termed as significant, while a ^∗∗^*p* < 0.01 is considered highly significant.

## 3. Results

### 3.1. XGC Reduced Cr and BUN Levels

**Figure 1:**
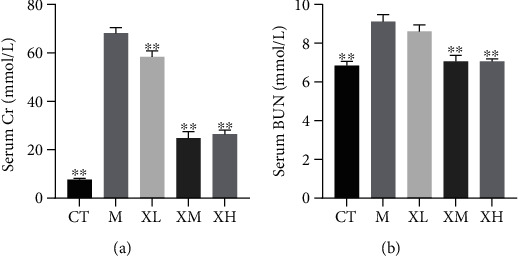
Effect of XGC on serum renal function index in mice. (a) Serum creatinine levels of mice in each group. (b) Serum urea nitrogen levels of mice in each group. (Note: CT-control group, M-model group, XL-XGC low-dose group, XM-XGC middle-dose group, and XH-XGC high-dose group).

An experimental model was established to further verify the mechanism of XGC in the treatment of kidney injury. The results showed that after 4 weeks of administration, the levels of serum urea nitrogen and creatinine in the model group were higher than those in the normal group (^∗∗^*p* < 0.01; Figure 1(b)), indicating that the renal injury model was established successfully. After the treatment of XGC, the results showed that the creatinine of XGC in low, middle, and high dose groups decreased averagely (^∗∗^*p* < 0.01), and the level of urea nitrogen decreased in the XGC middle group and high dose group (^∗∗^*p* < 0.01; Figure 1(a)).

### 3.2. XGC Treatment Mitigated Histopathological Damages

**Figure 2:**
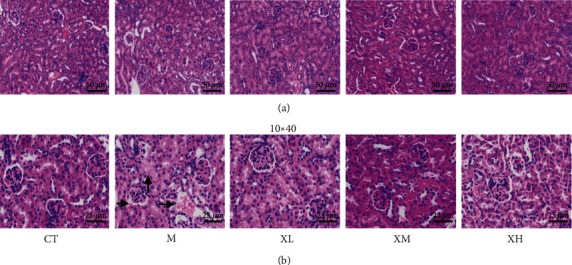
Effect of XGC on nephron in mice. (a) Observation of the kidney under a 200× microscope. (b) Observation of the kidney under a 400× microscope.

In the histological analysis, the renal tissue of the normal group showed that the morphology of glomeruli and tubules was normal, and there was no obvious tissue damage. Obvious tissue damage was found in the kidney tissue of mice in the model group, including diffuse edema and necrosis of renal tubules, dilatation, exudation of red blood cells, and glomerular atrophy and deformation. All dosage groups of XGC can reverse the injury of renal tissue (Figure 2).

### 3.3. XGC Protected the Damaged Kidney

**Figure 3:**
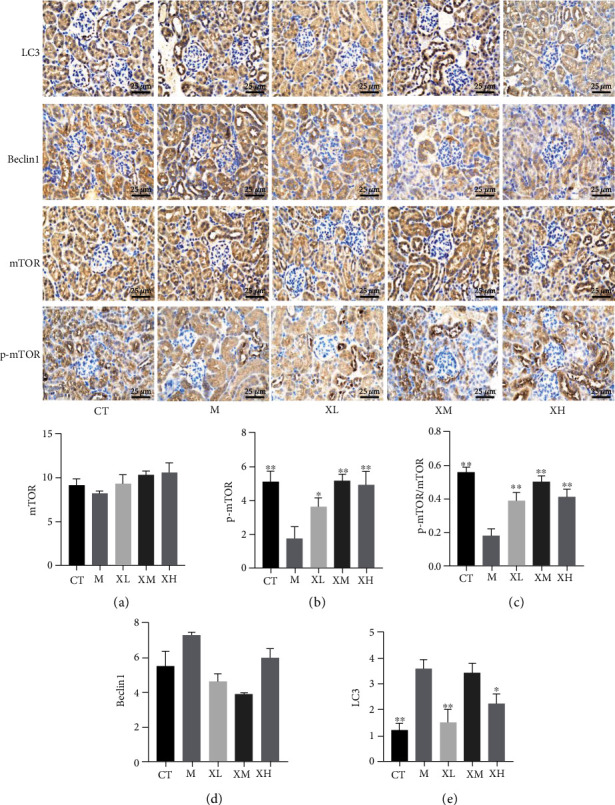
Effect of XGC on the protein for autophagy in kidney of mice. (a) The expression level of mTOR protein in each group. (b) The expression level of p-mTOR protein in each group. (c) The ratio of p-mTOR/mTOR in each group. (d) The expression level of Beclin1 protein in each group. (e) The expression level of LC3 protein in each group. (Note: CT-control group, M-model group, XL-XGC low-dose group, XM-XGC middle-dose group, and XH-XGC high-dose group).

**Figure 4:**
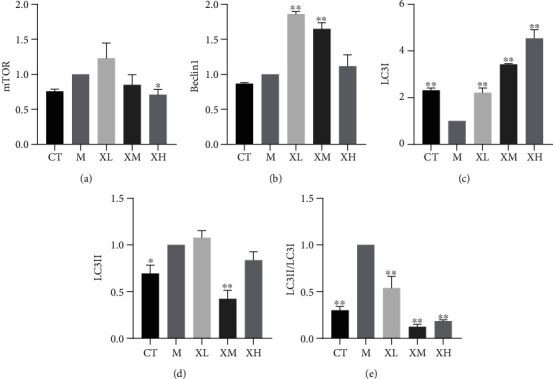
Effect of XGC on the mRNA for autophagy in kidney of mice. (a) The expression level of mTOR in each group. (b) The expression level of Beclin1 in each group. (c) The expression level of LC3I in each group. (d) The expression level of LC3II protein in each group. (e) The ratio of LC3II/LC3I in each group. (Note: CT-control group, M-model group, XL-XGC low-dose group, XM-XGC middle-dose group, and XH-XGC high-dose group).

**Figure 5:**
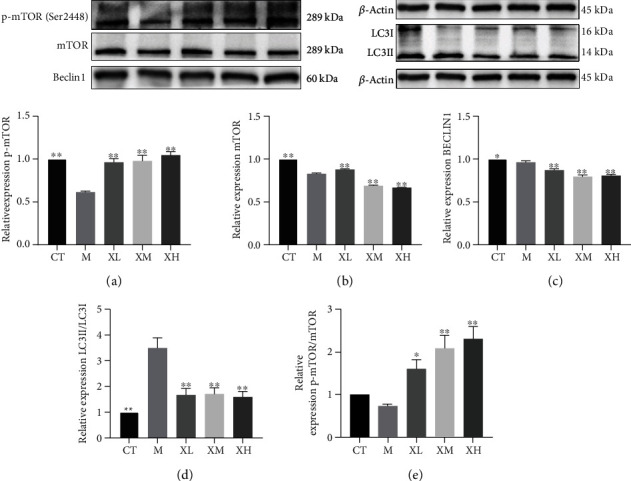
Effect of XGC on the protein for autophagy in kidney of mice. (a) The expression level of p-mTOR (Ser2448) in each group. (b) The expression level of mTOR in each group. (c) The expression level of Beclin1 in each group. (d) The ratio of LC3II/LC3I in each group. (e) The ratio of p-mTOR/mTOR in each group. (Note: CT-control group, M-model group, XL-XGC low-dose group, XM-XGC middle-dose group, and XH-XGC high-dose group).

As Figure 3 indicated, compared with the normal group, the expression of the p-mTOR protein in the model group was significantly decreased (^∗∗^*p* < 0.01), while the expression level of p-mTOR protein was significantly upregulated in low (^∗^*p* < 0.05), medium, and high (^∗∗^*p* < 0.01) dose group of XGC compared with the model group (Figure 3(a)). The ratio of p-mTOR/mTOR was significantly decreased (^∗∗^*p* < 0.01) in the model group compared with the normal group, and it was significantly increased in the low, medium, and high dose groups (^∗∗^*p* < 0.01) of XGC (Figure 3(c)). Compared with the normal group, the expression of LC3 protein in the model group was significantly increased, while the expression level of LC3 protein XGC was significantly downregulated in the low and the high dose group (^∗∗^*p* < 0.01, Figure 3(e)). As Figure 4 indicated, the gene expression of Beclin1 was significantly upregulated in the low (^∗∗^*p* < 0.01) and medium (^∗∗^*p* < 0.01) dose group of XGC compared with the model group (Figure 4(b)). And the gene expression of LC3I was significantly inhibited in the model group compared with the control group (^∗∗^*p* < 0.01), and all dosage groups (^∗∗^*p* < 0.01) of XGC were upregulated compared with the model group (Figure 4(c)). Compared with the control group, the ratio of LC3II/LC3I in the model group increased significantly (^∗∗^*p* < 0.01), and compared with the model group, the ratio of XGC in each dose group (^∗∗^*p* < 0.01) decreased significantly (Figure 4(e)). As Figure 5 indicated, the protein expression of Beclin1 was significantly upregulated in the low (^∗∗^*p* < 0.01), medium (^∗∗^*p* < 0.01), and high dose group of XGC compared with the model group (Figure 5(c)). Compared with the control group, the ratio of LC3II/LC3I in the model group increased significantly (^∗∗^*p* < 0.01), and compared with the model group, the ratio of XGC in each dose group (^∗∗^*p* < 0.01) decreased significantly (Figure 5(d)). The ratio of p-mTOR/mTOR was decreased (*p* > 0.05) in the model group compared with the normal group, and it was significantly increased in the low, medium, and high dose groups (^∗∗^*p* < 0.01) of XGC (Figure 5(e)).

## 4. Discussion

Kidney injury is a common disease with a long treatment period. Kidney injury can be caused by a variety of factors, including viral infections, autoimmune diseases, and heavy metal poisoning. As a heavy metal, cadmium is closely related to human life, and the accumulation of cadmium in the kidney will cause renal toxicity and aggravate the risk of CKD [15, 16]. So far, many drugs have been developed for the treatment of this disease. The “Kidney governing bone” theory plays a very important role in the long-term clinical practice of TCM, and experts have treated renal hypofunction with the technique of “tonifying kidney.” But the kidney-tonifying effect of XGC has never been studied. In this study, we investigated the preventive and protective effects of XGC on cadmium-induced kidney injury. Further research on the kidney injury experimental model in this study showed that the XGC could attenuate the kidney injury in model mice. And the treatment could reduce BUN and Cr levels and reverse the histopathological damage of the kidney. The results of this study suggested that the XGC has a preventive and therapeutic effect on kidney injury, by attenuating kidney tubules damage.

XGC is a Chinese medicine compound preparation, and every herb it contains plays a role in the protection of kidney injury. Epimedii Folium in traditional Chinese medicine has the effect of invigorating the kidney and strengthening yang. The icariin contained in Epimedii Folium has antioxidative stress, anti-inflammatory, and antiapoptotic effects, and it protects kidney 293 cells by inhibiting the ROS-mediated PI3K/Akt pathway [17]. Salvia miltiorrhiza Radix Et Rhizoma has a strong free radical scavenging ability and antioxidant properties, and the ethanol extract protects the kidney against ischemia-reperfusion damage [18]. Furthermore, studies have shown that Salvia miltiorrhiza Radix Et Rhizoma can restore the abnormal function of the kidney in iron-loaded mice and improve the pathological changes of the kidney in iron overload mice, and these remarkable protective effects are attributed to the prevention of inhibition of lipid peroxidation and nephrocyte apoptosis [19]. A study on gentamicin-induced kidney injury in rats showed that Anemarrhenae Rhizoma has a certain contribution to promote the proliferation of rat renal tubular cells and inhibit apoptosis [20]. Dipsaci Radix as a traditional Chinese medicine for the bone disease has been used in clinical practice for many years. In recent years, studies have found that the polysaccharides contained in it can improve the kidney injury caused by ischemia and reperfusion by removing active oxygen and increasing the activity of antioxidant enzymes [20]. The phenolic compounds in Psoraleae Fructus have high antioxidant activity. Studies have shown that bakuchiol can improve acute kidney injury caused by sepsis and improve the survival of patients [21]. Studies have shown that the roots and leaves of the traditional Chinese medicine Rehmanniae Radix have a certain ameliorating effect on the early kidney injury of diabetic mice, and it may be achieved by inhibiting the activation of the AGEs/RAGE/SphK signaling pathway [22, 23]. These results suggested that the XGC has a potent effect on kidney injury, by attenuating oxidative stress and nephrocyte apoptosis, and the bioactive compounds from the herbs of XGC may have a strong contribution to this efficacy.

When the human body is exposed to Cd, the kidney is the main organ affected. After Cd enters the body, it induces metallothionein (MT) to bind to it and forms the Cd-metallothionein complex (Cd-MT). Cd-MT is transported to the kidney through blood, and after glomerular filtration, it enters the renal tubule through pinocytosis, then is separated by lysosomes in the renal tubular cells and releases free Cd, which eventually deposits in the kidney, resulting in kidney injury [24]. After cadmium exposure, most of it accumulates in renal tubular epithelial cells [25]. Under the light microscope, the kidney of cadmium poisoning mice showed swelling and necrosis of renal tubular epithelial cells and irregular glomerular structure [25]. BUN and Cr are two common indexes for clinical evaluation of renal function. The increase of BUN indicates renal parenchyma lesion. Cr increases when glomerular filtration reduces [26]. This study showed that there were obvious edema, necrosis, and erythrocyte exudation of renal tubules in mice induced by CdCl_2_, and the damage of renal tubules was improved after preadministration of XGC in mice. Furthermore, BUN and Cr were increased in mice induced by CdCl_2_ and decreased in mice that were pretreated with XGC. This suggests that XGC can prevent and improve cadmium-induced renal injury, protect damaged renal tubules, and play a positive role in maintaining the normal physiological function of the nephron.

Autophagy is a stress response of cells to a variety of adverse environmental signals, such as hypoxia, oxidative stress, and nutritional depletion, which is usually related to cell protection or survival [27]. Autophagy can be divided into three types, including macrophage, microautophagy, and chaperone-mediated autophagy [28]. Unlike apoptosis leading to cell death, the function of autophagy has a dual nature. Under low-dose and short-term cadmium toxicity, autophagy, as a self-protection mechanism during cell damage, usually occurs in the early stage of kidney injury, while the level of autophagy in the later stage of the injury decreases, and even the protective mechanism turns to death mechanism. Cd can induce protective autophagy of renal tubular epithelial cells in the early stage of exposure [29–31]. In this study, intraperitoneal injection of CdCl_2_ for 30 days caused edema and necrosis of renal tubules in mice, and autophagy was also concentrated in renal tubules, which was consistent with previous studies, indicating that the model in this study was in the early stage of injury, and a kind of self-protective autophagy is taking place in the renal tubule.

The nutrition sensor mTOR is the core regulatory factor of autophagy [32, 33]. The mTOR plays a negative feedback regulation role in the process of autophagy [34]. Many factors upstream will activate or inhibit the activity of mTOR. Under physiological conditions, mTOR is in an activated state, and the expression level of p-mTOR is high, which inhibits the downstream autophagy pathway. When the body is in the early stage of injury, mTOR activity will be inhibited, thereby activating the downstream autophagy pathway to make the body in a self-protective state. The downstream autophagy process is divided into two stages. The first stage is the formation of the autophagosome membrane. The formed double-layer membrane envelops the cell components to be degraded and transports them to the lysosome. When autophagosomes bind to lysosomes, the second stage of autophagy is triggered [28]. The formation of autophagosomes is the center of the autophagy process [28]. Beclin-1 is a key factor regulating the initial process of autophagosome formation, indicating the initial level of autophagosome formation [35]. Lysosomes play a vital role in the process of autophagy, and LC3 is used as a lysosome-related protein for autophagy detection [28]. This study used IHC, RT-qPCR, and western-blot analysis to detect the key factors of autophagy, and the results showed that alone CdCl_2_ was the first to activate autophagy by inhibiting the activity of mTOR. Meanwhile, results showed that the expression level of LC3 protein in the cadmium alone treatment group was significantly higher than that in the XGC treatment group, and the Beclin1 gene expression level in the XGC treatment group was significantly higher than that in the cadmium alone treatment group, and gene expression level of LC3II/LC3I was significantly lower than that in the cadmium alone treatment group. These results further proved that XGC treatment delayed the occurrence of kidney injury in mice and inhibited the development of autophagy to apoptotic state.

## 5. Conclusions

Our findings confirmed that XGC could significantly decrease the level of BUN and Cr and delayed the occurrence of kidney injury and the process of autophagy. These fully demonstrate that XGC has a specific preventive and ameliorating effect on cadmium-induced kidney injury. Among the three dose groups we selected, the high-dose group had better efficacy. This study enriched the connotation of the theory of tonifying kidneys and provided another new option for treating kidney damage caused by heavy metals.

## Figures and Tables

**Table 1:** Primers for quantitative real-time PCR.

Gene	Primer sequence (5′–3′)	Primer length (bp)
mTOR	F:ACCGTCCGCCTTCACAGATACC	22
	R:GCAGTCCGTTCCTTCTCCTTCTTG	24
Beclin1	F:AGGCAGTGGCGGCTCCTATTC	21
	R:TGAGGACACCCAGGCAAGACC	21
LC3I	F:GAGCGAGTTGGTCAAGATCATCCG	24
	R:CATAGATGTCAGCGATGGGTGTGG	23
LC3II	F:TGTGTCCACTCCCATCTCCGAAG	23
	R:CCATTGCTGTCCCGAATGTCTCC	23
GAPDH	F:TCTCCTGCGACTTCAACA	18
	R:TGTAGCCGTATTCATTGTCA	20

## Data Availability

The data used to support the findings of this study are available from the corresponding author upon request.
